# Potential geographic "hotspots" for drug-injection related transmission of HIV and HCV and for initiation into injecting drug use in New York City, 2011-2015, with implications for the current opioid epidemic in the US

**DOI:** 10.1371/journal.pone.0194799

**Published:** 2018-03-29

**Authors:** D. C. Des Jarlais, H. L. F. Cooper, K. Arasteh, J. Feelemyer, C. McKnight, Z. Ross

**Affiliations:** 1 Icahn School of Medicine at Mount Sinai, New York, NY, United States of America; 2 Department of Behavioral Sciences and Health Education, Rollins School of Public Health, Emory University, Atlanta, Georgia, United States of America; 3 ZevRoss Spatial Analysis, Ithaca, New York, United States of America; Centers for Disease Control and Prevention, UNITED STATES

## Abstract

**Objective:**

We identified potential geographic “hotspots” for drug-injecting transmission of HIV and hepatitis C virus (HCV) among persons who inject drugs (PWID) in New York City. The HIV epidemic among PWID is currently in an “end of the epidemic” stage, while HCV is in a continuing, high prevalence (> 50%) stage.

**Methods:**

We recruited 910 PWID entering Mount Sinai Beth Israel substance use treatment programs from 2011–2015. Structured interviews and HIV/ HCV testing were conducted. Residential ZIP codes were used as geographic units of analysis. Potential “hotspots” for HIV and HCV transmission were defined as 1) having relatively large numbers of PWID 2) having 2 or more HIV (or HCV) seropositive PWID reporting transmission risk—passing on used syringes to others, and 3) having 2 or more HIV (or HCV) seronegative PWID reporting acquisition risk—injecting with previously used needles/syringes. Hotspots for injecting drug use initiation were defined as ZIP codes with 5 or more persons who began injecting within the previous 6 years.

**Results:**

Among PWID, 96% injected heroin, 81% male, 34% White, 15% African-American, 47% Latinx, mean age 40 (SD = 10), 7% HIV seropositive, 62% HCV seropositive. Participants resided in 234 ZIP codes. No ZIP codes were identified as potential hotspots due to small numbers of HIV seropositive PWID reporting transmission risk. Four ZIP codes were identified as potential hotspots for HCV transmission. 12 ZIP codes identified as hotspots for injecting drug use initiation.

**Discussion:**

For HIV, the lack of potential hotspots is further validation of widespread effectiveness of efforts to reduce injecting-related HIV transmission. Injecting-related HIV transmission is likely to be a rare, random event. HCV prevention efforts should include focus on potential hotspots for transmission and on hotspots for initiation into injecting drug use. We consider application of methods for the current opioid epidemic in the US.

## Introduction

Identifying potential geographic “hotspots” where there is a high likelihood of transmission of infectious diseases is a fundamental task of epidemiology. Such hotspots may develop in areas in which there is mixing of infectious and uninfected but susceptible persons. Knowledge of such hotspots can guide allocation of resources for the most effective and efficient efforts to reduce transmission.

In this report, we examine potential hotspots for drug injecting related transmission of HIV and hepatitis C virus (HCV) among persons who inject drugs (PWID) in New York City from 2011–2015. Both viruses can be transmitted through multi-person use (“sharing”) of needles and syringes, while PWID may additionally transmit HIV through high risk sexual behaviors. Hotspots for infectious disease transmission are typically studied through newly diagnosed infections [1–3]. This method can work well with diseases such as most sexually transmitted infections, for which the initial infection produces distinctive symptoms that lead the infected person to seek treatment and in geographic areas where it is unlikely that many susceptible persons are actively using prevention. Newly identified cases of drug injecting related HIV and HCV have been used for identifying potential geographic hotspots for further transmission [4–8], but these have important limitations. The initial infections with HIV and HCV may be asymptomatic or mildly and non-specifically symptomatic, so that the first diagnoses are often made long after the infection occurs. Newly identified cases of HIV or HCV infection also do not provide any information on the numbers of persons in the potential hotspot who are not yet infected but may be susceptible and at risk for high infection, so that predicting onward transmission can be difficult.

We examined potential hotspots for HIV and HCV transmission among PWID in New York City through bio-behavioral studies of the combination of 1) potential transmission risk behavior by persons seropositive for each virus and 2) potential acquisition risk behavior by persons seronegative for each virus. Combining both potential transmission risk and potential acquisition risk should provide greater precision for identifying potential hotspots than studying each type of risk separately.

The HIV and HCV epidemics among PWID in New York City are in quite different stages. HIV prevalence peaked at 50% to 60% among PWID in the early 1980s [9]. Since the implementation of multiple prevention and care interventions including needle/syringe exchange programs, medication assisted treatment, condom promotion, and antiretroviral treatment for seropositives, HIV incidence among PWID has now declined to an estimated incidence of 0.1/100 person-years (PY)[10] in New York City, and we now have reached an “end of the HIV epidemic” stage among PWID [11]. HCV prevalence was extremely high—approximately 90%—among PWID in New York City prior to the implementation of the HIV prevention interventions. HCV prevalence has since stabilized at approximately 70%, While the transmission of HCV among PWID in rural areas of the country is now receiving well-deserved attention [12, 13], HCV transmission also continues in urban areas [14–16]. Given the large numbers of PWID in urban areas [17] it is likely that there are considerably more new HCV infections occurring in urban areas than in rural areas. For example, HCV incidence among persons who begin injecting in New York City was recently estimated to be approximately 20/100 PY [18].

The New York State and City Departments of Health have current initiatives to “end the epidemics” of HIV and HCV in the State [19], and identification of potential hotspots may both provide measurement of progress and help target limited resources.

“New injectors,” defined as persons who have been injecting for short periods of time, are particularly likely to be exposed to HCV, with incidence rates of up to 20/100 person-years. New injectors are also particularly likely to recruit others into drug injecting [20]. We therefore sought to identify “new injector” hotspots which could serve as foci for both HCV prevention efforts and efforts to reduce transitions from non-injecting to injecting drug use.

We also consider application of our methods to the current opioid epidemic in the US.

## Materials and methods

The data presented here were collected as part of a long-running research study of persons entering Mount Sinai Beth Israel drug detoxification and methadone maintenance programs in New York City. The methods for this “Risk Factors” study have been previously described [9, 21] so only a summary will be presented here. The programs serve New York City as a whole and there were no changes in the requirements for entrance into the program over the study period.

In the detoxification program, research staff visited the general admission wards of the program in a preset order and examined all intake records of a specific ward to construct lists of patients admitted within the prior 3 days. All of the patients on the list for the specific ward were asked to participate in the study. As there was no relationship between the assignment of patients to wards and the order that the staff rotated through the wards, these procedures should produce an unbiased sample of persons entering the detoxification program. In the methadone program, newly admitted patients (those admitted in the previous month) were asked to participate in the research.

Participants were paid $20 for their time and effort. In both programs, approximately 95% of those asked agreed to participate. Common reasons for non-participation included medical appointments or other scheduled activities that would not permit study completion in a single visit.

Written informed consent was obtained and a trained interviewer administered a computer-assisted structured questionnaire covering demographics, drug use, risk behavior, and use of HIV prevention services. The questions on drug use and risk behavior referred to the 6 months prior to the interview, a time when the participants were not in substance use treatment. Thus, with respect to drug use and risk behavior, the participants should be considered a “prior to treatment” sample and not an “in treatment” sample. With respect to the participants’ residence, we asked “What is the ZIP code where you have slept the most during the last six months?”

Participants were then seen by counselors for HIV pretest counseling and serum collection. HIV testing was conducted at the New York City Department of Health laboratory using a commercial, enzyme-linked, immunosorbent assays (EIA) test with Western blot confirmation (BioRad Genetic Systems HIV-1-2+0 EIA and HIV-1 Western Blot, BioRad Laboratories, Hercules, CA). HCV testing was also conducted at the New York City Department of Health laboratory, using the Abbott HCV enzyme immunoassay (EIA) 2.0 test.

For the analyses presented here, only persons who reported having injected drugs at least once in the 6 months prior to program entry are included.

Subjects were permitted to participate on multiple occasions, though only once per calendar year. For these analyses, however, we utilized only the first interview for persons who participated multiple times in the study during the 2011–2015 period.

In this study, we examined ZIP codes in New York City as potential hotspots for HIV and HCV transmission among PWID. There were multiple reasons for selecting ZIP codes as the geographic unit of analysis. There were sufficient numbers of participants in the different ZIP codes to permit statistical analyses and to protect participant confidentiality. The New York State Department of Health has mapped New York City ZIP codes onto the different neighborhoods in New York City, (see https://www.health.ny.gov/statistics/cancer/registry/appendix/neighborhoods.htm). The population density of New York City is great enough that multiple ZIP codes map onto individual neighborhoods.

We defined potential hotspots as those ZIP codes meeting all 3 of the following criteria:

1. The ZIP code had to have a relatively large number of PWID, such that there were at least 10 participants in the study who reported residing in the ZIP code during the 6-month period prior to their interview; and
2. At least 2 respondents seropositive for HIV or HCV residing in the ZIP code had to report potential injecting-related risk for transmitting HIV or HCV, defined as passing on needles and syringes that they had used to other PWID (“distributive sharing”); and
3. At least 2 respondents not currently infected with HIV or HCV living in the ZIP code had to report potential injecting-related risk for acquiring HIV or HCV, defined as injecting with needles and syringes that had been used by other PWID (“receptive sharing”).

Note that the differential injecting risk behavior is towards transmission of the viruses—distributive sharing by seropositive persons and receptive sharing by seronegative persons—receptive sharing by seropositives and distributive sharing by seronegatives would not lead to transmission.

Persons who have recently begun injecting drugs often exhibit high rates of injecting risk behaviors and very high rates of acquisition of HCV [22]. After the first several years of injecting, HCV incidence typically declines due to saturation of high risk subgroups and reductions in injecting risk behavior [23]. Because New York City has been experiencing relatively large numbers of persons beginning to inject heroin, we generated potential “new injector” hotspots for HCV transmission and for initiating new persons into injecting drug use. These were operationally defined simply as ZIP codes with 5 or more “new injectors” (persons who had begun injecting within the previous 5 years). We did not use HCV serostatus differential risk behavior in the operational definition of new injector HCV hotspots because of the likelihood of rapid changes in both HCV serostatus (acquisition of HCV) and changes in risk behavior (toward risk reduction) during the period of being a “new injector.”

Stata software [24] was used for statistical analyses. The study was approved by the Mount Sinai Beth Israel Institutional Review Board.

## Results

### Residence, demographics, drug use and injecting risk behaviors

**Fig 1:**
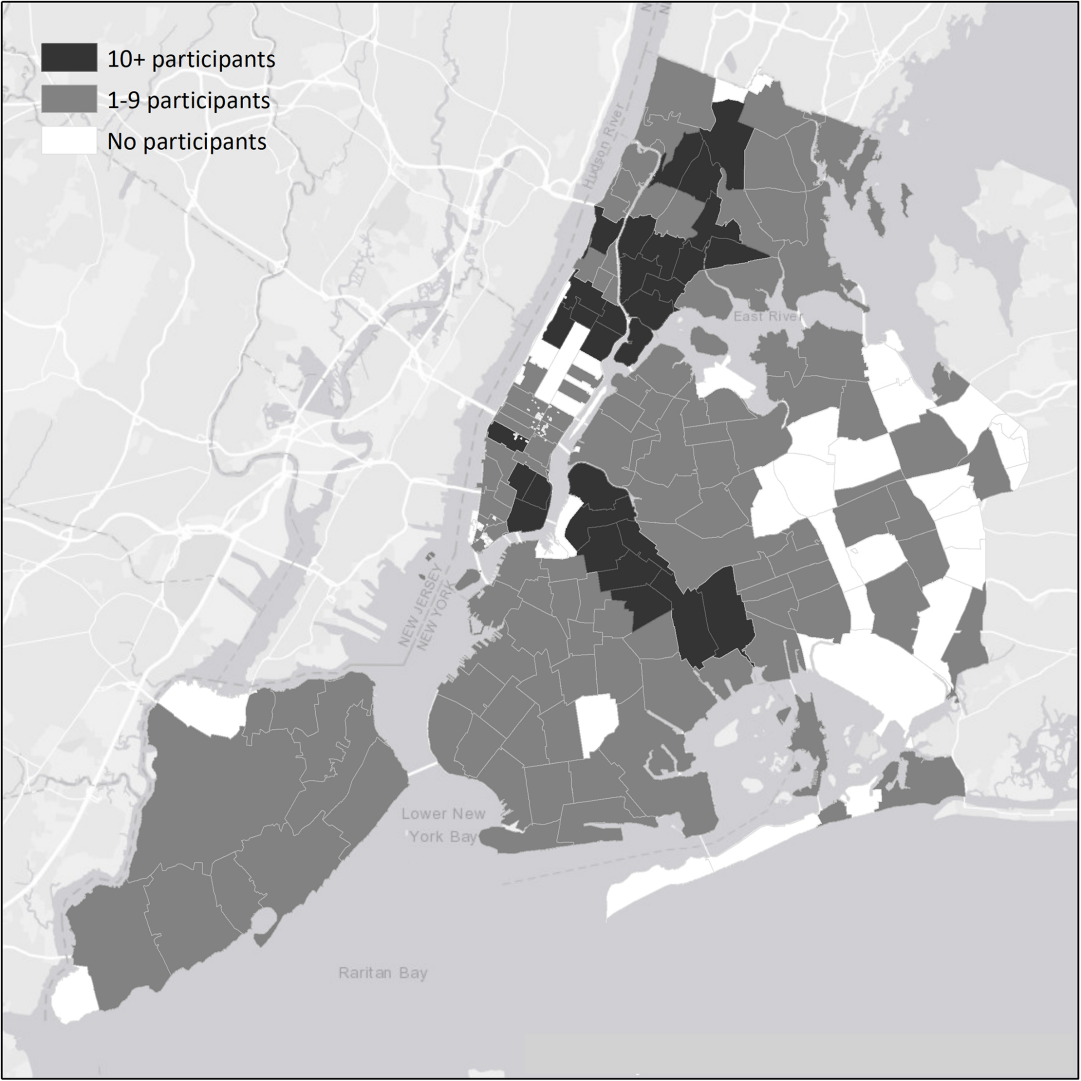
Number of participants by ZIP code in the 5 boroughs of New York City.

Fig 1 shows the ZIP codes in New York City in which our 910 study participants resided. These are categorized into ZIP codes that had relatively few (1–9) participants versus ZIP codes that had relatively many (10 or more) participants. The PWID in this study reside in a large number of ZIP codes in New York City, 144 of the total of 176 NYC ZIP codes, but there are concentrations in the “traditional” high drug use areas of New York City: Lower East Side and Harlem in Manhattan, central Brooklyn, and the South Bronx [25]. (See https://www.health.ny.gov/statistics/cancer/registry/appendix/neighborhoods.htm for a classification of ZIP codes into “neighborhoods” prepared by the New York State Department of Health.)

All participants injected drugs, 96% injected heroin, 81% were male, 34% were White, 15% were African-American, 47% were Latinx, the mean age was 40 (SD = 10), 7% were HIV seropositive and 62% were HCV seropositive. Table 1 presents the demographic characteristics, drug use behaviors, HIV and HCV prevalence, and HIV and HCV injecting risk behavior for study participants who resided in ZIP codes with 1–9 and 10 or more participants. There are many strong similarities, e.g., in drug use and in injecting risk behaviors, but also some notable differences. White participants were more likely to live in ZIP codes with 1–9 participants, while Latinx participants were more likely to reside in ZIP codes with 10 or more participants. A higher percentage of participants who reported smoking crack cocaine resided in ZIP codes with 1–9 participants. HIV and HCV prevalence were both significantly higher in ZIP codes with 10 or more participants. As expected the average number of study participants per ZIP code was much higher in the ZIP codes with 10 or more participants (mean of 17 participants per ZIP code) than in the ZIP codes with 1–9 participants (mean of 2 participants per ZIP code).

**Table 1:** Demographics and recent drug use of PWIDs in ZIP Codes with 10 or more PWIDs and less than 10 PWIDs who entered Beth Israel/Mount Sinai drug treatment from 2011–2015 (N = 910).

	Zips of 10 or more PWID		Zips of <10 PWID		All Zips	
Average N per Zip Code	17		2		4	
Average Age (SD)	41 (9.9)		38 (10.7)		40 (10.4)	
	N	%	N	%	N	%
Total	482	100.0	428	100.0	910	100
Gender						
Male	402	83.4	335	78.3	737	81.0
Female	80	16.6	93	21.7	173	19.0
Race/ethnicity*						
White	116	24.1	193	45.1	309	34.0
African-American	75	15.6	60	14.0	135	14.8
Latinx	281	58.3	145	33.9	426	46.8
Other	10	2.0	30	7.0	40	4.4
Age groups						
18–22	15	3.1	22	5.1	37	4.1
23–27	30	6.2	60	14.0	90	9.9
28–32	54	11.2	72	16.8	126	13.8
33–37	73	15.1	61	14.3	134	14.7
38–42	76	15.8	61	14.3	137	15.1
43–47	95	19.7	59	13.8	154	16.9
48–52	74	15.4	50	11.7	124	13.6
53–57	47	9.8	29	6.8	76	8.4
58–62	13	2.7	10	2.3	23	2.5
63–67	4	0.8	1	0.2	5	0.5
68–72	1	0.2	2	0.5	3	0.3
73–77	0	0.0	1	0.2	1	0.1
Injected heroin	456	94.8	414	96.7	870	95.7
Injected cocaine	188	39.1	171	40.1	359	39.6
Injected speedball	174	36.1	163	38.2	337	37.1
Smoked cocaine	182	37.8	187	43.7	369	40.6
Daily injection	346	71.8	313	73.1	659	72.4
HIV+*	41	9.8	16	4.2	57	7.2
HCV+*	277	66.4	216	57.0	493	61.9
ART among HIV+	26/41	63.4	10/16	62.5	36/57	63.2
HIV- with receptive sharing	68	14.2	71	16.9	139	15.5
HIV+ with distributive sharing	4	0.8	1	0.2	5	0.6
HCV- with receptive sharing	20	4.2	28	6.7	48	5.3
HCV+ with distributive sharing	42	8.9	34	8.0	76	8.5

* statistically significant differences, p < 0.05 by Fisher’s exact test

### Potential hotspots for HIV and HCV transmission

**Fig 2:**
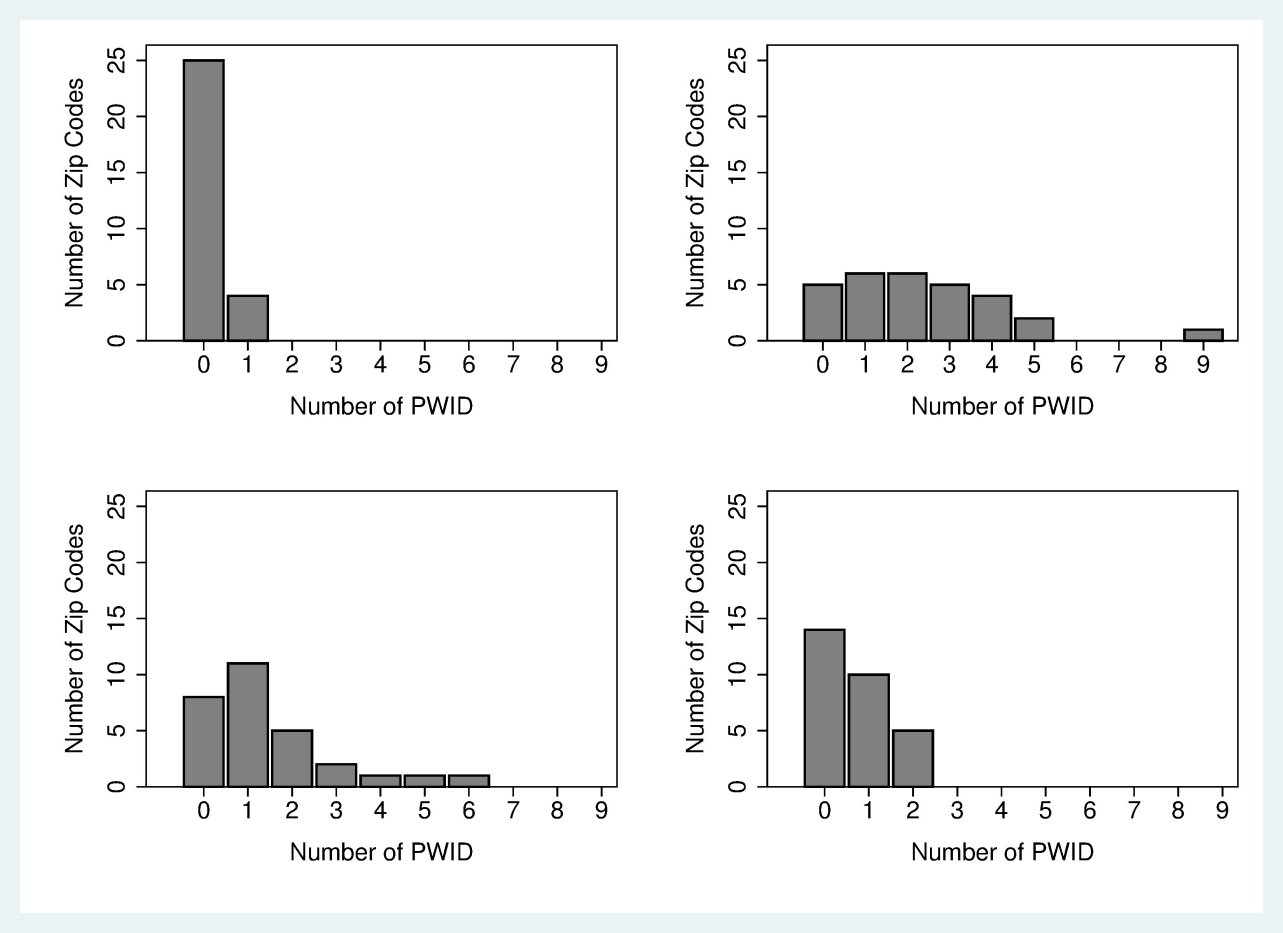
ZIP codes and distribution of HIV Positive PWID with distributive sharing; ZIP codes and distribution of HIV Negative PWID with receptive sharing; ZIP codes and distribution of HCV Positive PWID with distributive sharing; ZIP codes and distribution of HCV Negative PWID with receptive sharing.

**Fig 2** show the numbers of ZIP codes with different numbers of PWID reporting acquisition and transmission risk behaviors for HIV and HCV (among the 29 ZIP codes with 10 or more study participants). There was considerable variation in the numbers of ZIP codes with PWID reporting HIV and HCV injecting risk behaviors. For HIV, there were only 4 ZIP codes in which HIV seropositive PWID reported transmission risk behavior (distributive sharing) (Fig 2), while there were 24 ZIP codes in which HIV seronegative PWID reported acquisition risk behavior (receptive sharing) (Fig 2). For HCV, there were 21 ZIP codes in which HCV seropositive PWID reported transmission risk behavior (Fig 2), and 15 ZIP codes in which HCV seronegative PWID reported acquisition risk behavior (Fig 2). Overall, acquisition risk was more common for HIV and transmission risk was more common for HCV.

No ZIP codes met our above criteria for potential hotspots for HIV transmission because no ZIP code had at least 2 persons reporting HIV distributive sharing risk behavior. The 4 HIV seropositive participants who did report distributive sharing each resided in a different ZIP code. (Note also that 3 of these 4 PWID reported being on ART, so that these 3 would presumably be at reduced infectiousness.)

**Fig 3:**
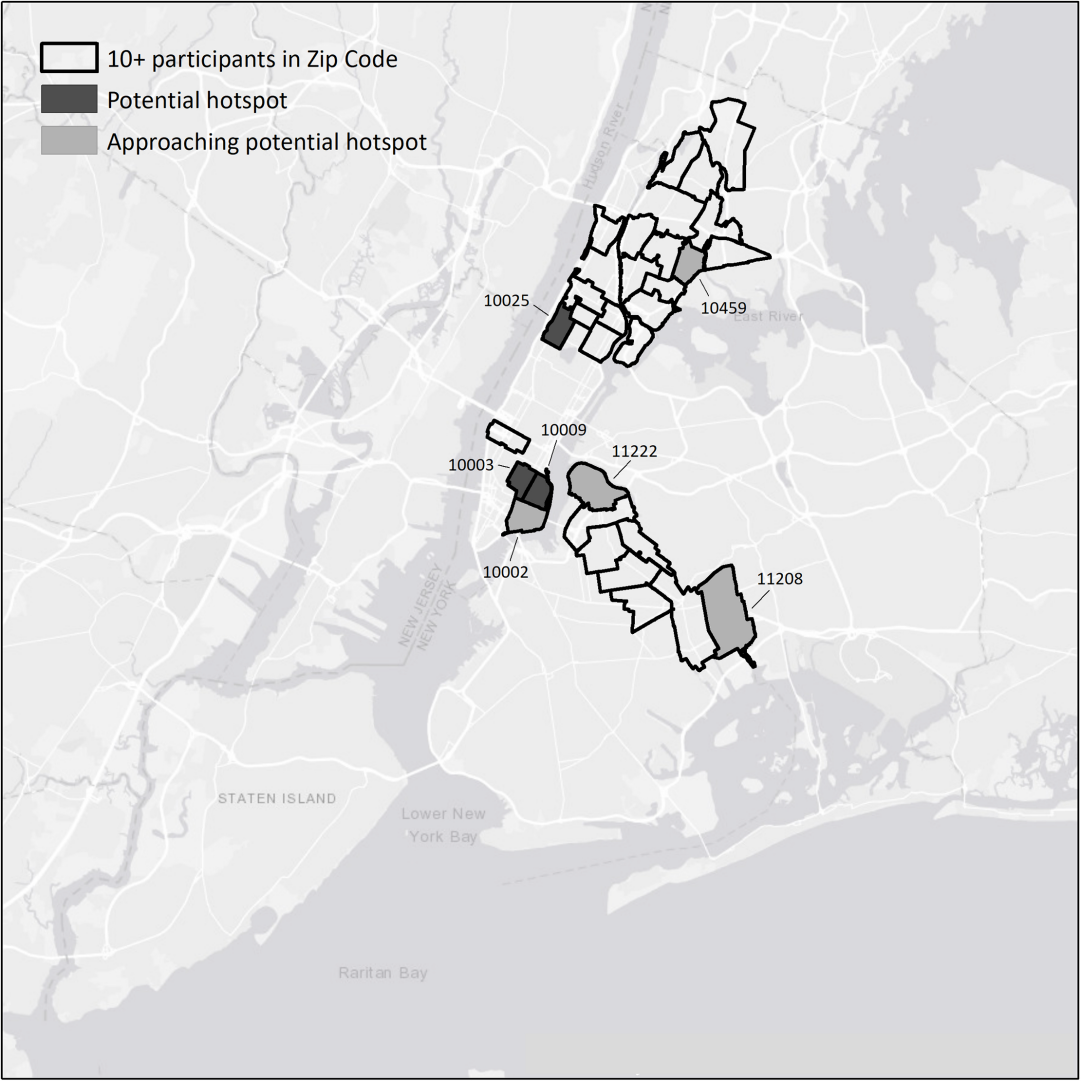
Potential hotspots for HCV transmission among PWID in the 5 boroughs of New York City.

As noted in the introduction, both HCV prevalence and estimated HCV incidence are much higher than HIV prevalence and estimated HIV incidence. Three ZIP codes (10003, 10009, and 10025 in Manhattan) met our definition of potential hotspot for HCV transmission. Four other ZIP codes, 10002 in lower Manhattan, 10459 in the Bronx, and 11208 and 11222 in Brooklyn approached our definition of a potential HCV hotspot, with 2 or more HCV seropositive participants reporting distributive sharing but only 1 HCV seronegative participant reporting receptive sharing. These ZIP codes are shown in Fig 3. Note, again, the cluster of ZIP codes on the Lower Eastside of Manhattan.

There was considerable variation in both the numbers of study participants in the ZIP codes with 10 or more participants and in the number of participants reporting injecting-related risk behaviors in these ZIP codes. We examined the correlations between the total number of participants in each ZIP code and the number of participants 1) reporting transmission risk for HCV, and 2) reporting acquisition risk for HCV. The correlation between total number of participants in each ZIP code and the number of HCV seropositive participants in the ZIP code reporting transmission risk was significant (r = 0.63, r^2^ = 0.40, p < 0.005); the correlation between the total number of participants in the ZIP code and the number of HCV seronegative participants reporting acquisition risk was not significant (r = 0.12, p = 0.5).

### Hotspots for initiation into injecting drug use

As noted above, persons who have recently begun injecting drugs are typically at very high risk for exposure to HCV [22]. There were 277 “new injectors” (persons who had been injecting for 5 or fewer years) and they comprised 30% of our total sample. There HIV prevalence was 3.2% (95% CI 1.4 to 6.1) and their HCV prevalence was 34.1% (95% CI 28.3 to 40.3). Table 2 presents HCV prevalence by years injecting in our total sample. There is a rapid increase in HCV prevalence for the first five years of injecting, corresponding to an HCV incidence rate of 15 to 20/100 person-years.

**Table 2:** HCV prevalence by years injecting.

Years injecting	Total N (%)	HCV + N (%)
< = 1	104 (100.0)	24 (23.1)
2–3	83 (100.0)	27 (32.5)
4–5	65 (100.0)	35 (53.9)
6–10	112 (100.0)	69 (61.6)
10–15	129 (100.0)	87 (67.4)
16+	303 (100.0)	251 (82.8)

**Fig 4:**
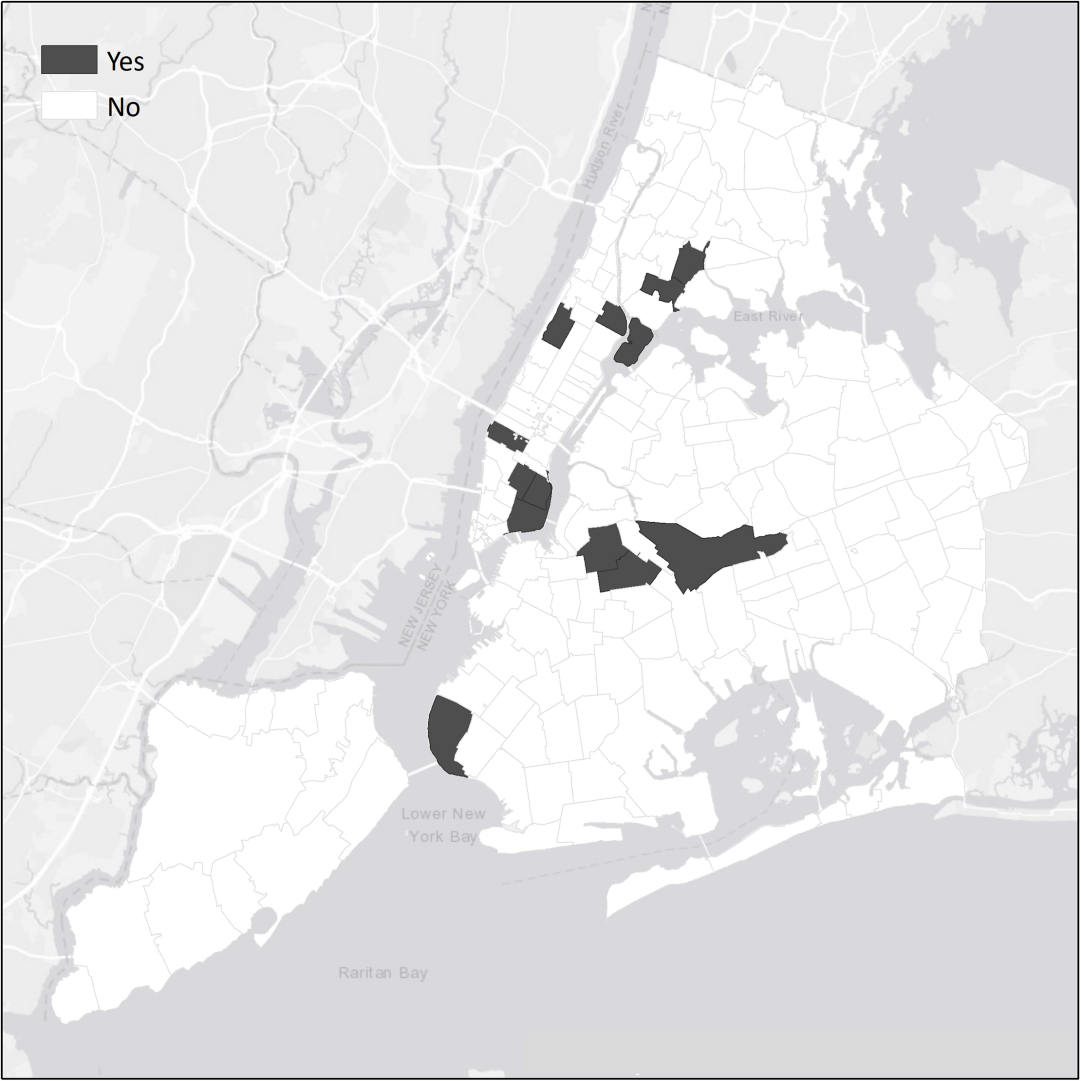
Potential initiation hotspots among PWID in the 5 boroughs of New York City.

We mapped potential new injector HCV hotspots as ZIP codes with 5 or more new injectors. Fig 4 shows the 12 or more “new injector hotspot” ZIP codes.

Note that all 3 of the ZIP codes that were classified as potential hotspots for HCV transmission are included in the new injector hotspots. The new injector hotspots also include ZIP codes for Randall’s Island (10035) and for the Chelsea/Clinton and Lower Eastside districts (10001 and 10002). All of these areas have multiple homeless shelters. The new injectors in these areas may include many “urban nomad”[26] homeless, young/new injectors who travel to New York City. It is also likely that these areas include many persons transitioning from non-injecting to injecting drug use.

## Discussion

In this study, we used a combination of biological data (HIV and HCV serostatus) and differential risk behavior data to identify potential ZIP code hotspots for the injecting-related transmission of HIV and HCV among PWID in New York City. To our knowledge, this is the first study to include both biological (HIV and HCV serostatus) and differential risk behavior data (distributive sharing among seropositives and receptive sharing among seronegatives) to examine potential hotspots for both HIV and HCV transmission among PWID.

Our data show both dispersion and concentration of PWID residences in New York City. Participants reported residing in 144 (80%) out of the 176 ZIP codes in New York City (see Fig 1). Injecting drug use clearly is not confined to only a few areas in New York City. More than half (482/910) of our participants, however, resided in the 29 ZIP codes that had at least 10 participants each. Moreover, PWID residing in these 29 ZIP codes had both higher HIV and HCV prevalence. Additionally, the number of PWID in each of these 29 ZIP codes was associated (r^2^ = 0.40) with the number of HCV seropositive PWID in the ZIP code who reported current distributive sharing of needles and syringes. We do not have the dates for actual infection with HIV or HCV among our participants—it is likely that almost all of the infections occurred before the 6-month period prior to the interview—but it would appear likely that living in an area with relatively few other PWID may be protective against acquiring HIV or HCV through syringe sharing. Future research should examine potential mechanisms through which higher concentrations of PWID within local geographic areas may lead to higher rates of adverse consequences of injecting drug use. This may be particularly relevant for understanding racial/ethnic disparities in adverse health outcomes related to psychoactive drug use.

We did not observe any ZIP codes that met our criteria for potential hotspots for injecting-related transmission of HIV. This was due to the combination of low HIV prevalence, low numbers of HIV seropositive PWID who reported distributive sharing, and the geographic dispersion of the HIV seropositive participants who did report distributive sharing across different ZIP codes. The lack of potential hotspots in our data for HIV transmission is consistent with the current very low incidence of HIV among PWID in New York City (estimated at 0.1/100 person-years) and reflects decades of high coverage combined HIV prevention and care for PWID in New York City[27]. Notably, the New York State Department of Health AIDS Institute currently has an initiative to eliminate injecting-related transmission of HIV in the state. While it may be difficult to prevent all injecting-related transmission of HIV, the data in this report suggest that if the public-health scale interventions in New York City can be replicated throughout the state, injecting-related HIV transmission could be reduced to very infrequent semi-random events. (Two major issues for replication of the New York City scale of interventions throughout the state are delivering high coverage in small city and rural areas and the increase in injecting drug use due to the new opioid epidemic in many parts of the state.)

In this report we examined potential geographic hotspots for injecting related transmission of HIV and HCV among PWID. PWID are also vulnerable to sexual transmission of HIV, and modeling and HCV/HSV-2 studies suggest that the majority of new HIV infections among PWID in New York City are due to sexual transmission [28]. Potential hotspots for sexual transmission of HIV among PWID and among non-injecting drug users (NIDUs) will be examined in a separate paper.

We did observe ZIP code hotspots for continuing transmission of HCV. These ZIP codes were in areas long potential known to have high concentrations of injecting drug use, including the Lower Eastside, the South Bronx, and Central Brooklyn. Previous research on HCV transmission in New York City has shown particularly high HCV incidence in the Lower Eastside.[29, 30]

We also observed 12 ZIP code “new injector” hotspots that included homeless shelters and also where transitions from non-injecting to injecting drug use may be occurring. We believe that this study is the first to assess such hotspots with a sample that includes PWID from throughout the city. These new injector hotspots would be appropriate for interventions to reduce HCV transmission and for efforts to reduce initiation into injecting. Many of the PWID residing in these areas are likely to require multiple health and social services.

“Combined prevention and care” including needle/syringe exchange programs, medication-assisted substance use treatment, and HIV treatment as prevention has been very successful in reducing HIV transmission in New York City, and should be intensified to reduce HCV transmission. The relatively high number of HCV seropositive PWID who engaged in distributive sharing was a major driver of potential hotspots for HCV transmission. Additional research on factors contributing to distributive syringe sharing among HCV seropositive PWID is urgently needed.

The overarching conceptual question guiding this study was to identify potential geographic areas (operationalized as ZIP codes) for continued HIV and/or HCV transmission among PWID in New York City. For HIV, the high coverage of evidence-based prevention and care intervention appears to have successfully eliminated such potential hotspots. For HCV transmission, our data show considerable evidence for the importance of geography:

1. Even though injecting drug use is present in many parts of New York City, injecting drug use is concentrated in a modest number of ZIP codes.
2. HCV prevalence is significantly higher among PWID who reside in ZIP codes with larger numbers of PWID.
3. There is great variation in the numbers of HCV seropositive PWID engaging in distributive sharing among the ZIP codes with relatively large numbers of PWID (see Table 2). A substantial proportion of this variability can be explained simply by the numbers of PWID residing in these ZIP codes (r^2^ = 0.40), but it is highly likely that there are other geographically organized factors that contribute to distributive sharing by HCV seropositive PWID. Such factors may include economic disadvantage, demographic composition, access to syringe service and substance use treatment programs, police activities that interfere with safer injection practice, and access to treatment for HCV infection [31–35]
4. Potential HCV transmission through mixing of HCV seropositive PWID engaging in distributive syringe sharing and HCV seronegative PWID engaging in receptive syringe sharing is concentrated in a relatively small number of ZIP codes.
5. New injectors (persons injecting for 5 years or less) are concentrated in a modest number of ZIP codes.

### Implications for the current “opioid epidemic” in the US

The US is currently experiencing an opioid epidemic with high rates of overdoses, new injecting drug use, new HCV infections and potential new HIV infections [36–39]. The CDC has identified counties at particularly high risk for these public health problems [40]. The methods used in this report combine data on demographic characteristics HIV/HCV serostatus, differential injecting risk behavior, and length of time since first injection. Having such data in areas experiencing opioid epidemics could be extremely useful for targeting resources for substance use treatment, overdose prevention and reversal, and for HCV/HIV prevention. We would recommend consideration of adaptations of these methods to identify potential HIV and HCV outbreaks in areas currently experiencing opioid epidemics. As many of the current opioid epidemics are occurring in suburban and rural areas of the US, adaptation of our methods will involve a number of factors:

1. We collected data from a service provider, and many current opioid epidemics are occurring in small city/rural areas that lack services for PWID. Clearly additional services will need to be provided in opioid epidemic areas and systematic data collection should be conducted from persons utilizing those services. Community-based data collection would also be highly useful.
2. Our data were collected and then analyzed over a period of years, but available electronic records and secure internet communications could permit close to real time data collection and analyses in opioid epidemic areas.
3. We used face-to-face interviewing, but self-interviewing (probably tablet based) should permit more efficient data collection where limited staff are available. We also used serum for HIV and HCV testing, but rapid testing would provide for easier specimen collection and immediate provision of results.
4. Our current data analyses are limited to injecting-related HIV and HCV transmission and initiation into injecting drug use, but data elements to address overdose could be easily added.
5. We used ZIP codes as our geographic unit of analyses; ZIP codes were developed to facilitate mail delivery and have many limitations for studying relationships between neighborhood characteristics and health outcomes. As noted above, the New York City Health Department has been able to map ZIP codes to neighborhoods, but such mapping would be difficult in locations with low population density, and different geographic units would need to be used in suburban and rural areas.

In this report, we have used combined biological and differential injecting risk behavior data to further corroborate the “end of the HIV epidemic” among PWID in New York City and to identify potential hotspots for continuing HCV transmission among PWID in the city. Integrating biological and behavioral data (for both transmission and acquisition risk) might provide a means for limiting the potential public health problems in the emerging opioid epidemics in the US.

### Limitations

Several limitations of this study should be noted. First, the participants were recruited from entrants into a single system of substance use programs. This is the largest substance use treatment system in New York City, and as shown in Fig 1, our study participants come from many different areas of the city. HIV infection among entrants into this treatment system has tracked consistently with HIV infection data from other sources in New York City [10, 41–45]. Most importantly, there is close agreement between HIV incidence measured in the Risk Factors study and estimated HIV incidence in data from the New York City Department of Health and Mental Hygiene HIV Surveillance unit [10].

“New injectors” who have not yet engaged in any substance use treatment could not have been recruited into this study. We can, however, compare data on our new injectors with data from a recently published study of “young” injectors in New York City. Eckhardt and colleagues [14] recruited 714 “young” (ages 18 to 35) people who inject drugs to a storefront research site. Recruitment occurred through community outreach and “snowball” sampling, in which current participants are asked to recruit new participants. Eckhardt et al. found a sharp increase in HCV prevalence by years injecting (see their Table 2 and Fig 1) similar to that in our data. (See our Table 2). They found an HCV prevalence of 48% (95% CI 44.4 to 51.7), while among the 298 participants aged 18 to 35 in this study HCV prevalence was 43.2% (95% CI 37.6 to 49.1). The data on young/new injectors in this study are quite consistent with the data on community-recruited young/new injectors in the Eckhardt et al. study.

Second, we used ZIP codes as our geographic unit of analysis. ZIP codes have varying numbers of residents and the boundaries of ZIP codes in most cities do not necessarily match well with neighborhoods. However, the New York State Department of Health has mapped ZIP codes onto neighborhoods (see https://www.health.ny.gov/statistics/cancer/registry/appendix/neighborhoods) and this mapping appears to capture neighborhoods with large numbers of drug users quite well.

Third, we used the ZIP codes in which our study participants resided. PWID may also inject drugs and engage in risk behavior outside of the ZIP codes in which they reside. We suspect that injecting outside of one’s residential area would, however, be most likely to occur in the areas traditionally known for drug distribution (Lower Eastside, Harlem, South Bronx, Central Brooklyn) which our analyses did identify as potential hotspots for continuing HCV transmission. Additionally, even if persons inject outside of the ZIP code in which they reside, it may be helpful to provide HIV and HCV prevention services near where they live [46].

Fourth, we have not identified the social determinants of health or the causal mechanisms that generated the geographic distributions in the prevalence of injecting drug use, of HIV and HCV among PWID, or of injecting risk behaviors. Additional research will be required to identify these mechanisms and to identify the most effective interventions for countering these factors.

These limitations are important, but would not appear to have artificially generated the patterns in our data. Rather, we believe it is likely that the patterns would emerge despite these limitations.

## Conclusions

In studying the distribution of risks for drug injecting related risks for HIV and HCV transmission among PWID in New York City, we did not observe any current potential hotspots for continuing injecting-related transmission of HIV. We identified three potential hotspots for continuing transmission of HCV and 12 potential hotspots for initiation into injection. Additional research is needed to determine the causal mechanisms that generate these geographic distributions. High coverage implementation of evidence-based prevention and care interventions has led to an “end of the HIV epidemic” among PWID in New York City [10]. The political will behind the dramatic reduction of HIV among PWID, and the lessons learned from this experience, now need to be applied to HCV. To this end, combining biological data, differential risk behavior data, injection initiation data (new injectors) and geographic location data could enhance attempts to greatly reduce HCV transmission in the current opioid epidemic in the US.

## Supporting information

S1 Appendix List of Zip codes by neighborhood, New York City five boroughs (Bronx, Brooklyn, Manhattan, Queens, and Staten Island. (PDF)

S2 Appendix Zip codes by borough Maps. (PDF)

## References

[1] Dowdy DW, Golub JE, Chaisson RE, Saraceni V. Heterogeneity in tuberculosis transmission and the role of geographic hotspots in propagating epidemics. Proceedings of the National Academy of Sciences. 2012;109(24):9557–62. 10.1073/pnas.1203517109 PMC3386125 22645356

[2] Levy MZ, Bowman NM, Kawai V, Plotkin JB, Waller LA, Cabrera L, et al Spatial patterns in discordant diagnostic test results for Chagas disease: links to transmission hotspots. Clin Infect Dis. 2009 4 15;48(8):1104–6. doi: 10.1086/597464 19278335 10.1086/597464 PMC2766414

[3] Paull SH, Song S, McClure KM, Sackett LC, Kilpatrick AM, Johnson PT. From superspreaders to disease hotspots: linking transmission across hosts and space. Front Ecol Environ. 2012 Mar 1;10(2):75–82. doi: 10.1890/110111 23482675 10.1890/110111 PMC3589764

[4] Kauhl B, Heil J, Hoebe CJ, Schweikart J, Krafft T, Dukers-Muijrers NH. The spatial distribution of hepatitis C virus infections and associated determinants—An application of a geographically weighted poisson regression for evidence-based screening interventions in hotspots. PLoS One. 2015 9 9;10(9):e0135656 doi: 10.1371/journal.pone.0135656 26352611 10.1371/journal.pone.0135656 PMC4564162

[5] Meyers DJ, Hood ME, Stopka TJ. HIV and hepatitis C mortality in Massachusetts, 2002–2011: spatial cluster and trend analysis of HIV and HCV using multiple cause of death. PLoS One. 2014 12 11;9(12):e114822 doi: 10.1371/journal.pone.0114822 25502820 10.1371/journal.pone.0114822 PMC4263669

[6] Ramjee G, Wand H. Geographical clustering of high risk sexual behaviors in “hot-spots” for HIV and sexually transmitted infections in Kwazulu-Natal, South Africa. AIDS Behav. 2014 2;18(2):317–22. doi: 10.1007/s10461-013-0578-x 23934268 10.1007/s10461-013-0578-x PMC3905176

[7] Wand H, Ramjee G. Targeting the hotspots: investigating spatial and demographic variations in HIV infection in small communities in South Africa. J Int AIDS Soc. 2010 10 27;13:41 doi: 10.1186/1758-2652-13-41 20979651 10.1186/1758-2652-13-41 PMC2984578

[8] Boodram B, Golub ET, Ouellet LJ. Socio-behavioral and geographic correlates of prevalent hepatitis C virus infection among young injection drug users in metropolitan Baltimore and Chicago. Drug Alcohol Depend. 2010 9 1;111(1–2):136–45. doi: 10.1016/j.drugalcdep.2010.04.003 20472373 10.1016/j.drugalcdep.2010.04.003

[9] Des Jarlais DC, Friedman SR, Novick DM, Sotheran JL, Thomas P, Yancovitz S, et al HIV-1 infection among intravenous drug users in Manhattan, New York City, from 1977 through 1987. JAMA. 1989 2 17;261(7):1008–12. 2915408 10.1001/jama.261.7.1008

[10] Des Jarlais DC, Arasteh K, McKnight C, Feelemyer J, Campbell AN, Tross S, et al Consistent Estimates of Very Low HIV Incidence Among People Who Inject Drugs: New York City, 2005–2014. Am J Public Health. 2016 3;106(3):503–8. doi: 10.2105/AJPH.2015.303019 26794160 10.2105/AJPH.2015.303019 PMC4815962

[11] Des Jarlais DC, Arasteh K, McKnight C, Feelemyer J, Tross S, Perlman D, et al Racial/Ethnic Disparities at the End of an HIV Epidemic: Persons Who Inject Drugs in New York City, 2011–2015. Am J Public Health. 2017 7;107(7):1157–1163. doi: 10.2105/AJPH.2017.303787 28520494 10.2105/AJPH.2017.303787 PMC5463217

[12] Appalachian Regional Commission. Communicting about Opioids in Appalachia: Challenges, Opportunities and Best Practices. Tennessee: 2018.

[13] Fraser H, Zibbell J, Hoerger T, Hariri S, Vellozzi C, Martin NK, et al Scaling up HCV prevention and treatment interventions in rural USA–model projections for tackling an increasing epidemic. Addiction. 2018 1;113(1):173–182. doi: 10.1111/add.13948 28734093 10.1111/add.13948 PMC6211174

[14] Eckhardt B, Winkelstein ER, Shu MA, Carden MR, McKnight C, Des Jarlais DC, et al Risk factors for hepatitis C seropositivity among young people who inject drugs in New York City: Implications for prevention. PLoS One. 2017 5 19;12(5):e0177341 doi: 10.1371/journal.pone.0177341 28542351 10.1371/journal.pone.0177341 PMC5438142

[15] Morris MD, Shiboski S, Bruneau J, Hahn JA, Hellard M, Prins M, et al Geographic Differences in Temporal Incidence Trends of Hepatitis C Virus Infection Among People Who Inject Drugs: The InC3 Collaboration. Clin Infect Dis. 2017 4 1;64(7):860–869. doi: 10.1093/cid/ciw869 28362947 10.1093/cid/ciw869 PMC5439493

[16] Neaigus A, Reilly KH, Jenness SM, Hagan H, Wendel T, Gelpi-Acosta C, et al Trends in HIV and HCV Risk Behaviors and Prevalent Infection Among People Who Inject Drugs in New York City, 2005–2012. J Acquir Immune Defic Syndr. 2017 7 1;75 Suppl 3:S325–S332. doi: 10.1097/QAI.0000000000001407 28604434 10.1097/QAI.0000000000001407 PMC8284853

[17] Tempalski B, Pouget ER, Cleland CM, Brady JE, Cooper HL, Hall HI, et al Trends in the population prevalence of people who inject drugs in US metropolitan areas 1992–2007. PLoS One. 2013 6 5;8(6):e64789 doi: 10.1371/journal.pone.0064789 23755143 10.1371/journal.pone.0064789 PMC3673953

[18] Jordan AE, Des Jarlais DC, Arasteh K, McKnight C, Nash D, Perlman DC. Incidence and prevalence of hepatitis c virus infection among persons who inject drugs in New York City: 2006–2013. Drug Alcohol Depend. 2015 7 1;152:194–200. doi: 10.1016/j.drugalcdep.2015.03.039 25891230 10.1016/j.drugalcdep.2015.03.039 PMC4458155

[19] New York State Department of Health. For achieving the goal set forth by Governor Cuomo to end the epidemic in New York State by the end of 2020. Albany: 2015.

[20] Harocopos A, Goldsamt LA, Kobrak P, Jost JJ, Clatts MC. New injectors and the social context of injection initiation. Int J Drug Policy. 2009 7;20(4):317–23. doi: 10.1016/j.drugpo.2008.06.003 18790623 10.1016/j.drugpo.2008.06.003 PMC2706152

[21] Des Jarlais DC, Arasteh A, Hagan H, McKnight C, Perlman D, Friedman S. Persistence and change in disparities in HIV infection among injecting drug users in New York City after large-scale syringe exchange. Am J Public Health. 2009 10;99 Suppl 2:S445–51. doi: 10.2105/AJPH.2008.159327 19797757 10.2105/AJPH.2008.159327 PMC4451117

[22] Hagan H, Pouget ER, Des Jarlais DC. A systematic review and meta-analysis of interventions to prevent hepatitis C virus infection in people who inject drugs. J Infect Dis. 2011 7 1;204(1):74–83. doi: 10.1093/infdis/jir196 21628661 10.1093/infdis/jir196 PMC3105033

[23] Kuo M, Janjua NZ, Burchell AN, Buxton JA, Krajden M, Gilbert M. Decreasing Hepatitis C incidence among a population with repeated tests: British Columbia, Canada, 1993–2011. Am J Public Health. 2015 8;105(8):1604–10. doi: 10.2105/AJPH.2015.302591 26066920 10.2105/AJPH.2015.302591 PMC4504305

[24] STATA Corp. Stata 12. College Station, Texas2012.

[25] Courtwright D, Joseph H, Des Jarlais DC. Addicts Who Survived: An Oral History of Narcotic Use in America 1923–1965. Knoxville: University of Tennessee Press; 1989.

[26] Des Jarlais DC, Perlis T, Settembrino J. The use of electronic debit cards in longitudinal data collection with geographically mobile drug users. Drug Alcohol Depend. 2005 1 7;77(1):1–5 doi: 10.1016/j.drugalcdep.2004.06.010 15607835 10.1016/j.drugalcdep.2004.06.010

[27] Des Jarlais DC, Arasteh K, McKnight C, Feelemyer J, Hagan H, Cooper HL, et al Combined HIV Prevention, the New York City Condom Distribution Program, and the Evolution of Safer Sex Behavior Among Persons Who Inject Drugs in New York City. AIDS Behav. 2014 3;18(3):443–51. doi: 10.1007/s10461-013-0664-0 24271348 10.1007/s10461-013-0664-0 PMC3947424

[28] Des Jarlais DC, Arasteh K, McKnight C, Perlman DC, Feelemyer J, Hagan H, et al HSV-2 Co-infection as a driver of HIV transmission among heterosexual non-injecting drug users in New York City. PLoS One. 2014 1 31;9(1):e87993 doi: 10.1371/journal.pone.0087993 24498235 10.1371/journal.pone.0087993 PMC3909306

[29] Des Jarlais DC, Diaz R, Perlis T, Vlahov D, Maslow C, Latka M, et al Variability in the incidence of HIV, HBV, and HCV infection among young injecting drug users in New York City. Am J Epidemiol. 2003 3 1;157(5):467–71. 12615611 10.1093/aje/kwf222

[30] Diaz T, Des Jarlais DC, Vlahov D, Perlis TE, Edwards V, Freidman SR, et al Factors associated with prevalent hepatitus C virus infection: Differences among young adult injection drug users in lower and upper Manhattan, New York City. Am J Public Health. 2001 1;91(1):23–30. 11189819 10.2105/ajph.91.1.23 PMC1446499

[31] Bluthenthal RN, Kral AH, Gee L, Erringer EA, Edlin BR. The effect of syringe exchange use on high-risk injection drug users: a cohort study. AIDS. 2000 3 31;14(5):605–11. 10780722 10.1097/00002030-200003310-00015

[32] Davis C, Burris S, Kraut-Becher J, Lynch K, Metzger D. Effects of an intensive street-level police intervention on syringe exchange program use in Philadelphia, PA. Am J Public Health. 2005 2;95(2):233–6. doi: 10.2105/AJPH.2003.033563 15671455 10.2105/AJPH.2003.033563 PMC1449157

[33] De P, Cox J, Boivin JF, Platt RW, Jolly AM. The importance of social networks in their association to drug equipment sharing among injection drug users: a review. Addiction. 2007 11;102(11):1730–9. doi: 10.1111/j.1360-0443.2007.01936.x 17935581 10.1111/j.1360-0443.2007.01936.x

[34] Galea S, Vlahov D. Social Determinants and the Health of Drug Users: Socioeconomic Status, Homelessness, and Incarceration. Public Health Rep. 2002;117 Suppl 1:S135–45. 12435837 PMC1913691

[35] Sorensen JL, Copeland AL. Drug abuse treatment as an HIV prevention strategy: a review. Drug Alcohol Depend. 2000 4 1;59(1):17–31. 10706972 10.1016/s0376-8716(99)00104-0

[36] Peters PJ, Pontones P, Hoover KW, Patel MR, Galang RR, Shields J, et al HIV infection linked to injection use of oxymorphone in Indiana, 2014–2015. N Engl J Med. 2016 7 21;375(3):229–39. doi: 10.1056/NEJMoa1515195 27468059 10.1056/NEJMoa1515195

[37] Rudd RA, Aleshire N, Zibbell JE, Matthew Gladden R. Increases in drug and opioid overdose deaths—United States, 2000–2014. MMWR Morb Mortal Wkly Rep. 2016 1 1;64(50–51):1378–82. doi: 10.15585/mmwr.mm6450a3 26720857 10.15585/mmwr.mm6450a3

[38] Van Handel MM, Rose CE, Hallisey EJ, Kolling JL, Zibbell JE, Lewis B, et al County-level vulnerability assessment for rapid dissemination of HIV or HCV infections among persons who inject drugs, United States. J Acquir Immune Defic Syndr. 2016 11 1;73(3):323–331. doi: 10.1097/QAI.0000000000001098 27763996 10.1097/QAI.0000000000001098 PMC5479631

[39] Wilkerson RG, Kim HK, Windsor TA, Mareiniss DP. The opioid epidemic in the United States. Emerg Med Clin North Am. 2016 5;34(2):e1–e23. doi: 10.1016/j.emc.2015.11.002 27133253 10.1016/j.emc.2015.11.002

[40] O’Donnell JK GR, Seth P. Trends in Deaths Involving Heroin and Synthetic Opioids Excluding Methadone, and Law Enforcement Drug Product Reports, by Census Region—United States, 2006–2015. MMWR Morb Mortal Wkly Rep. 2017 9 1;66(34):897–903. doi: 10.15585/mmwr.mm6634a2 28859052 10.15585/mmwr.mm6634a2 PMC5657786

[41] Des Jarlais DC, Perlis T, Friedman SR, Chapman T, Kwok J, Rockwell R, et al Behavioral risk reduction in a declining HIV epidemic: injection drug users in New York City, 1990–1997. Am J Public Health. 2000 7;90(7):1112–6. 10897190 10.2105/ajph.90.7.1112 PMC1446283

[42] Des Jarlais DC, Perlis T, Friedman SR, Deren S, Chapman TF, Sotheran JL, et al Declining seroprevalence in a very large HIV epidemic: injecting drug users in New York City, 1991 to 1996. American journal of public health. 1998;88(12):1801–6. 9842377 10.2105/ajph.88.12.1801 PMC1509056

[43] Des Jarlais DC, Marmor M, Friedmann P, Aviles E, Deren S, Torian LV, et al HIV incidence among injecting drug users in New York City, 1992–1997: Evidence for a declining epidemic. Am J Public Health. 1998 12;88(12):1801–6. 10705851 10.2105/ajph.90.3.352 PMC1446171

[44] Murrill CS, Prevots DR, Miller MS, Linley LA, Royalty JE, Gwinn M. Incidence of HIV among injection drug users entering drug treatment programs in four US cities. J Urban Health. 2001 3;78(1):152–61. doi: 10.1093/jurban/78.1.152 11368194 10.1093/jurban/78.1.152 PMC3456197

[45] Thomas P. 25 years of HIV in New York City: Lessons from surveillance. J Urban Health. 2001 12;78(4):669–78. doi: 10.1093/jurban/78.4.669 11796813 10.1093/jurban/78.4.669 PMC3455868

[46] Rockwell R, Des Jarlais D, Friedman S, Perlis T, Paone D. Geographic Proximity, Policy and Utilization of Syringe Exchange Programmes. AIDS Care. 1999 8;11(4):437–42. doi: 10.1080/09540129947811 10533536 10.1080/09540129947811

