# Supplementary material for: Potential geographic "hotspots" for drug-injection related transmission of HIV and HCV and for initiation into injecting drug use in New York City, 2011-2015, with implications for the current opioid epidemic in the US
Source: PLoS One. 2018 Mar 29;13(3):e0194799. doi: 10.1371/journal.pone.0194799 (PMC5875800; doi:10.1371/journal.pone.0194799)
Supplement: S1 Appendix — (PDF) [file pone.0194799.s001.pdf]

List of Zip Codes by Neighborhood, New York City Five Boroughs (Bronx, Brooklyn, Manhattan, Queens, and Staten Island

**Bronx**	
Central Bronx	10453, 10457, 10460
Bronx Park and Fordham	10458, 10467, 10468
High Bridge and Morrisania	10451, 10452, 10456
Hunts Point and Mott Haven	10454, 10455, 10459, 10474
Kingsbridge and Riverdale	10463, 10471
Northeast Bronx	10466, 10469, 10470, 10475
Southeast Bronx	10461, 10462,10464, 10465, 10472, 10473
**Brooklyn**	
Central Brooklyn	11212, 11213, 11216, 11233, 11238
Southwest Brooklyn	11209, 11214, 11228
Borough Park	11204, 11218, 11219, 11230
Canarsie and Flatlands	11234, 11236, 11239
Southern Brooklyn	11223, 11224, 11229, 11235
Northwest Brooklyn	11201, 11205, 11215, 11217, 11231
Flatbush	11203, 11210, 11225, 11226
East New York and New Lots	11207, 11208
Greenpoint	11211, 11222
Sunset Park	11220, 11232
Bushwick and Williamsburg	11206, 11221, 11237
**Manhattan**	
Central Harlem	10026, 10027, 10030, 10037, 10039
Chelsea and Clinton	10001, 10011, 10018, 10019, 10020, 10036
East Harlem	10029, 10035
Gramercy Park and Murray Hill	10010, 10016, 10017, 10022
Greenwich Village and Soho	10012, 10013, 10014
Lower Manhattan	10004, 10005, 10006, 10007, 10038, 10280
Lower East Side	10002, 10003, 10009
Upper East Side	10021, 10028, 10044, 10065, 10075, 10128
Upper West Side	10023, 10024, 10025
Inwood and Washington Heights	10031, 10032, 10033, 10034, 10040
**Queens**	
Northeast Queens	11361, 11362, 11363, 11364
North Queens	11354, 11355, 11356, 11357, 11358, 11359, 11360
Central Queens	11365, 11366, 11367
Jamaica	11412, 11423, 11432, 11433, 11434, 11435, 11436
Northwest Queens	11101, 11102, 11103, 11104, 11105, 11106
West Central Queens	11374, 11375, 11379, 11385
Rockaways	11691, 11692, 11693, 11694, 11695, 11697
Southeast Queens	11004, 11005, 11411, 11413, 11422, 11426, 11427, 11428, 11429
Southwest Queens	11414, 11415, 11416, 11417, 11418, 11419, 11420, 11421
West Queens	11368, 11369, 11370, 11372, 11373, 11377, 11378
**Staten Island**	
Port Richmond	10302, 10303, 10310
South Shore	10306, 10307, 10308, 10309, 10312
Stapleton and St. George	10301, 10304, 10305
Mid-Island	10314
