# Supplementary material for: Potential geographic "hotspots" for drug-injection related transmission of HIV and HCV and for initiation into injecting drug use in New York City, 2011-2015, with implications for the current opioid epidemic in the US
Source: PLoS One. 2018 Mar 29;13(3):e0194799. doi: 10.1371/journal.pone.0194799 (PMC5875800; doi:10.1371/journal.pone.0194799)

Zip Codes Brooklyn New York

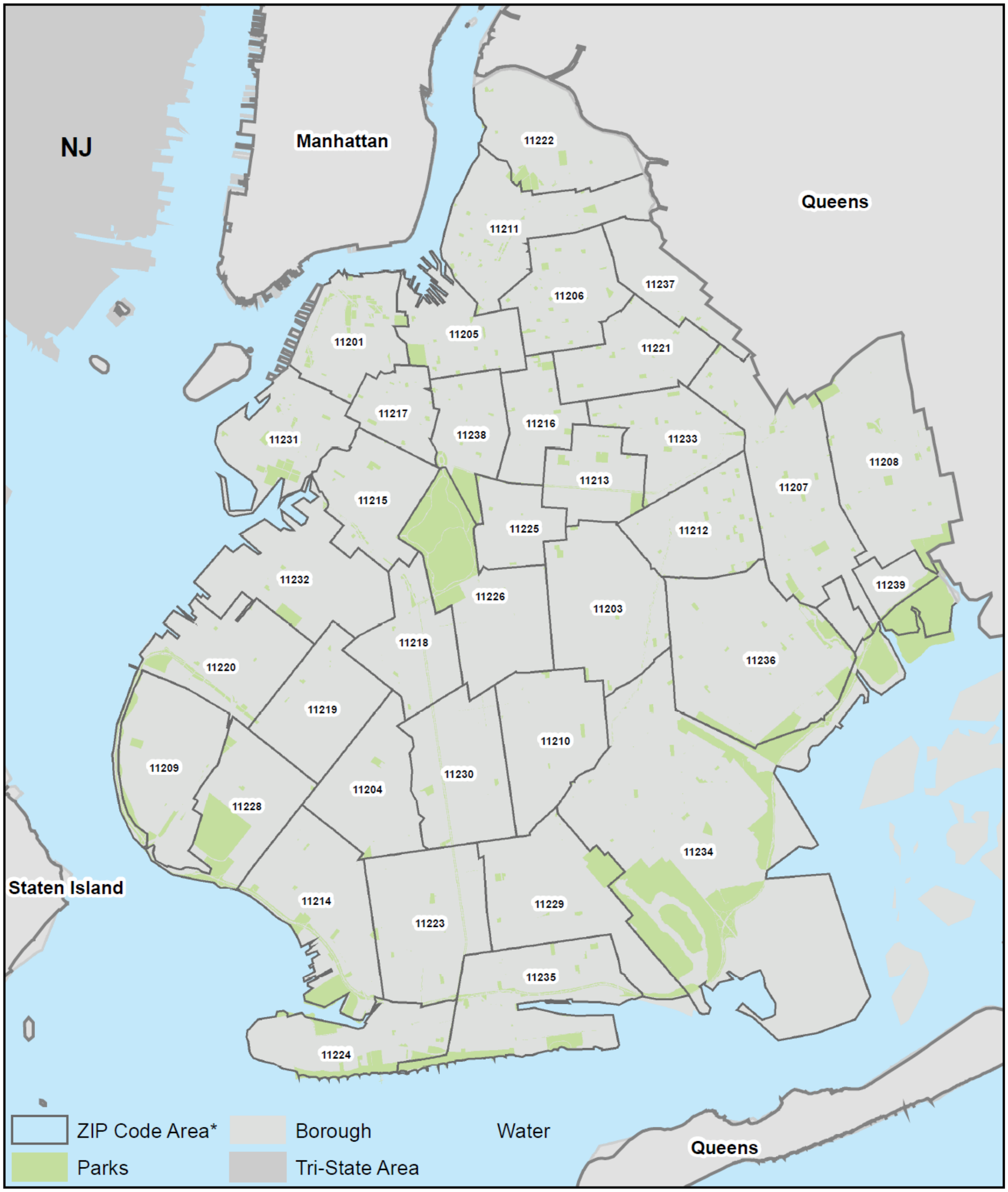

Zip Codes The Bronx New York

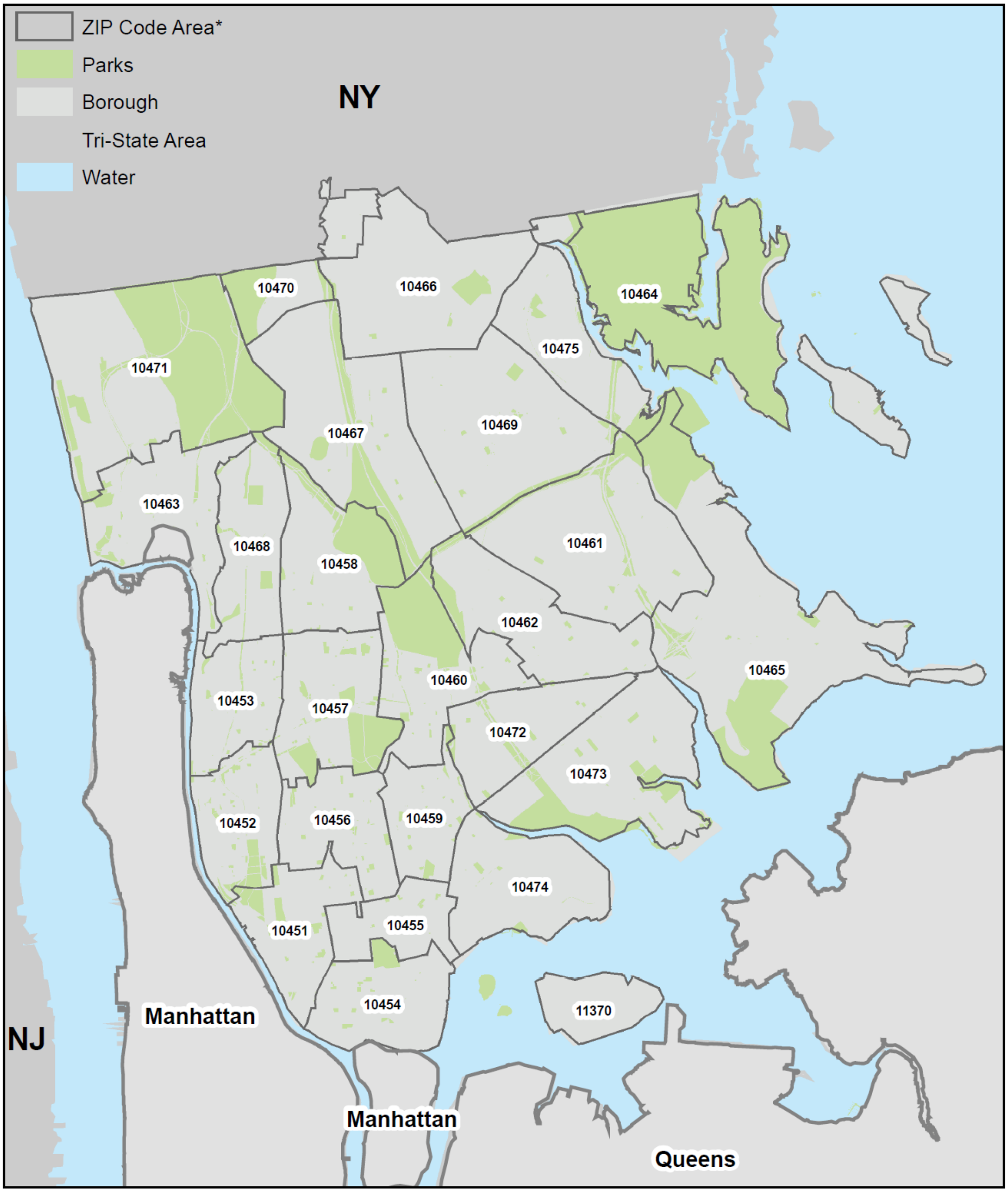

Zip Codes Manhattan New York

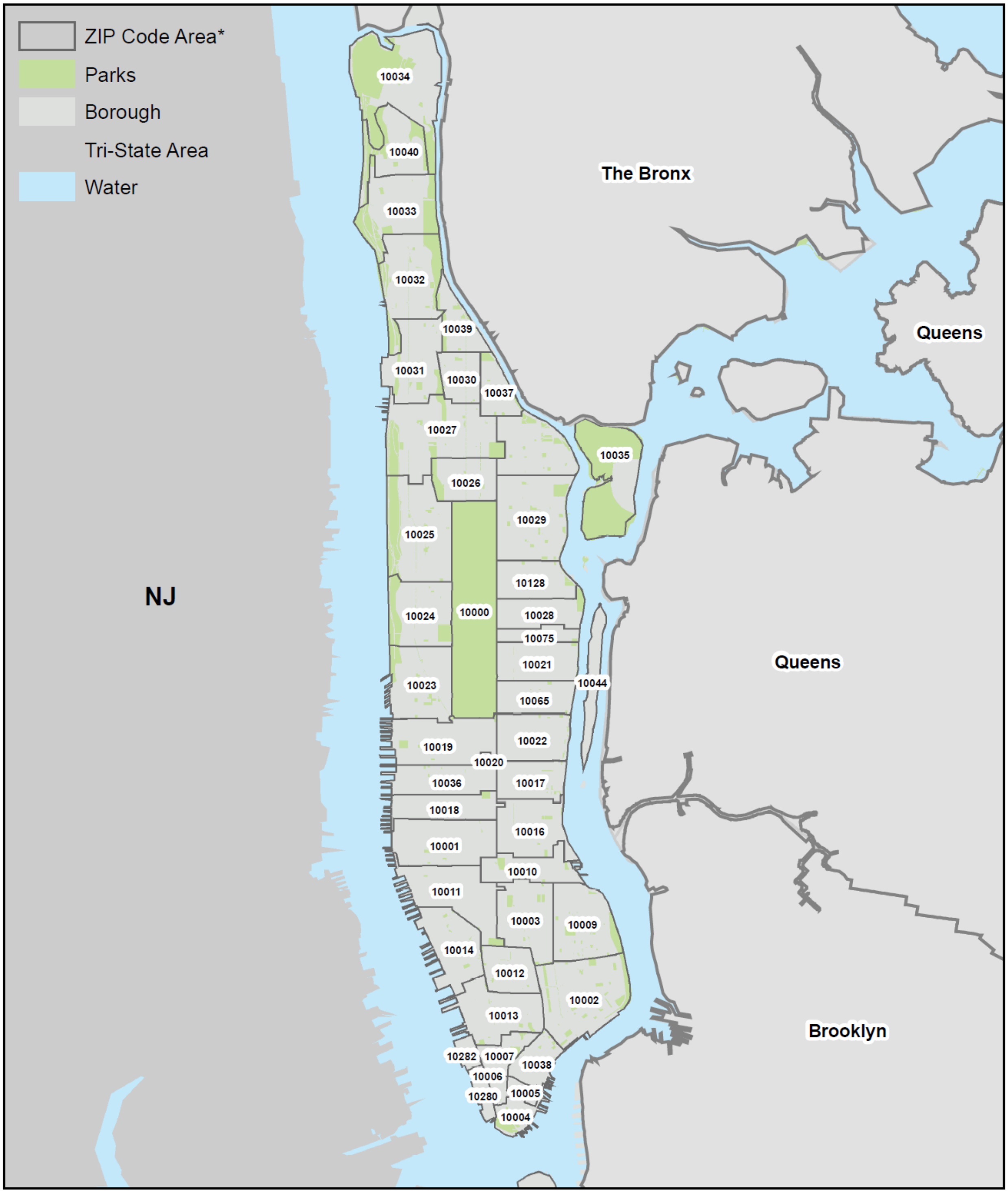

Zip Codes for Queens New York

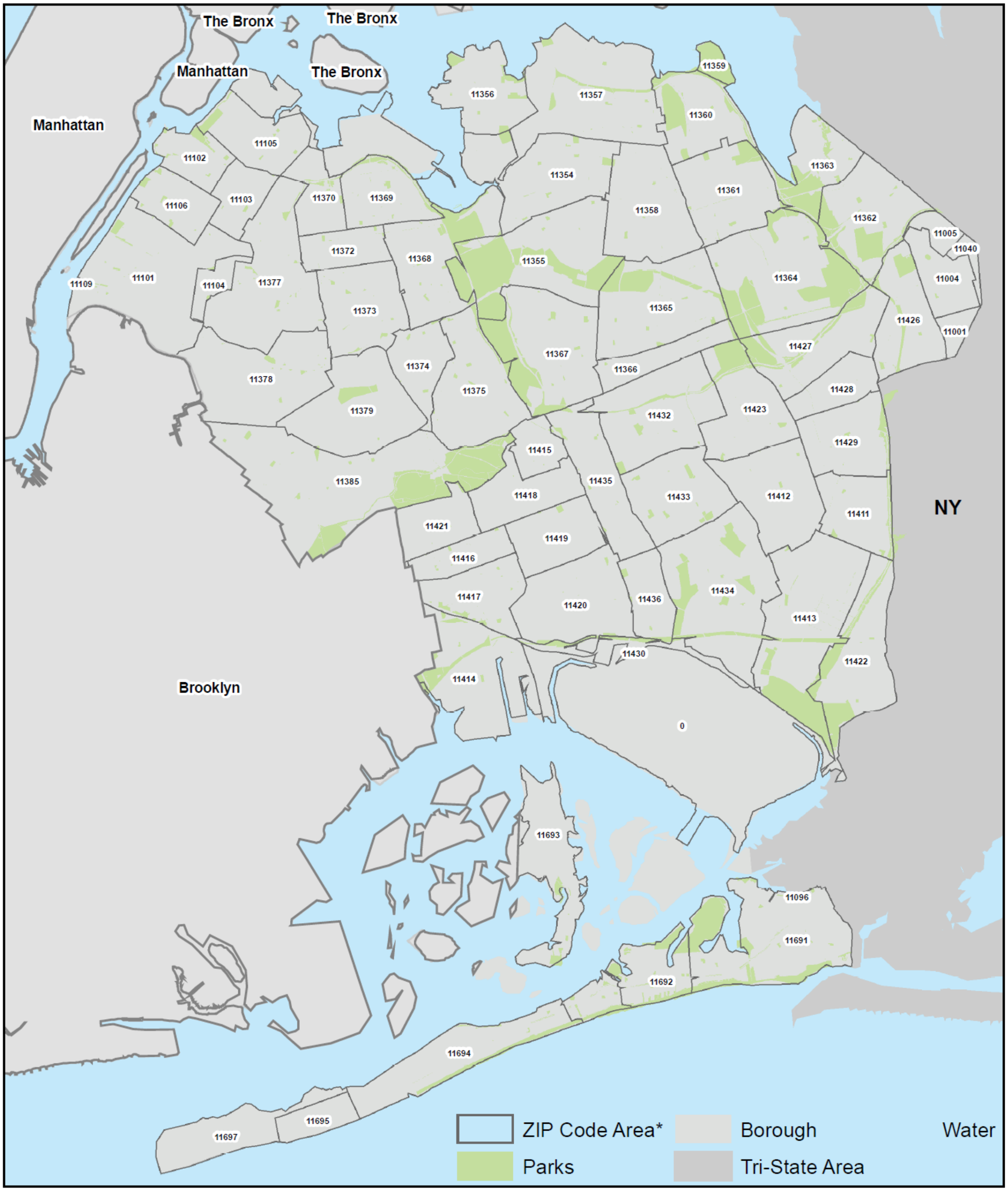

Zip Codes for Staten Island

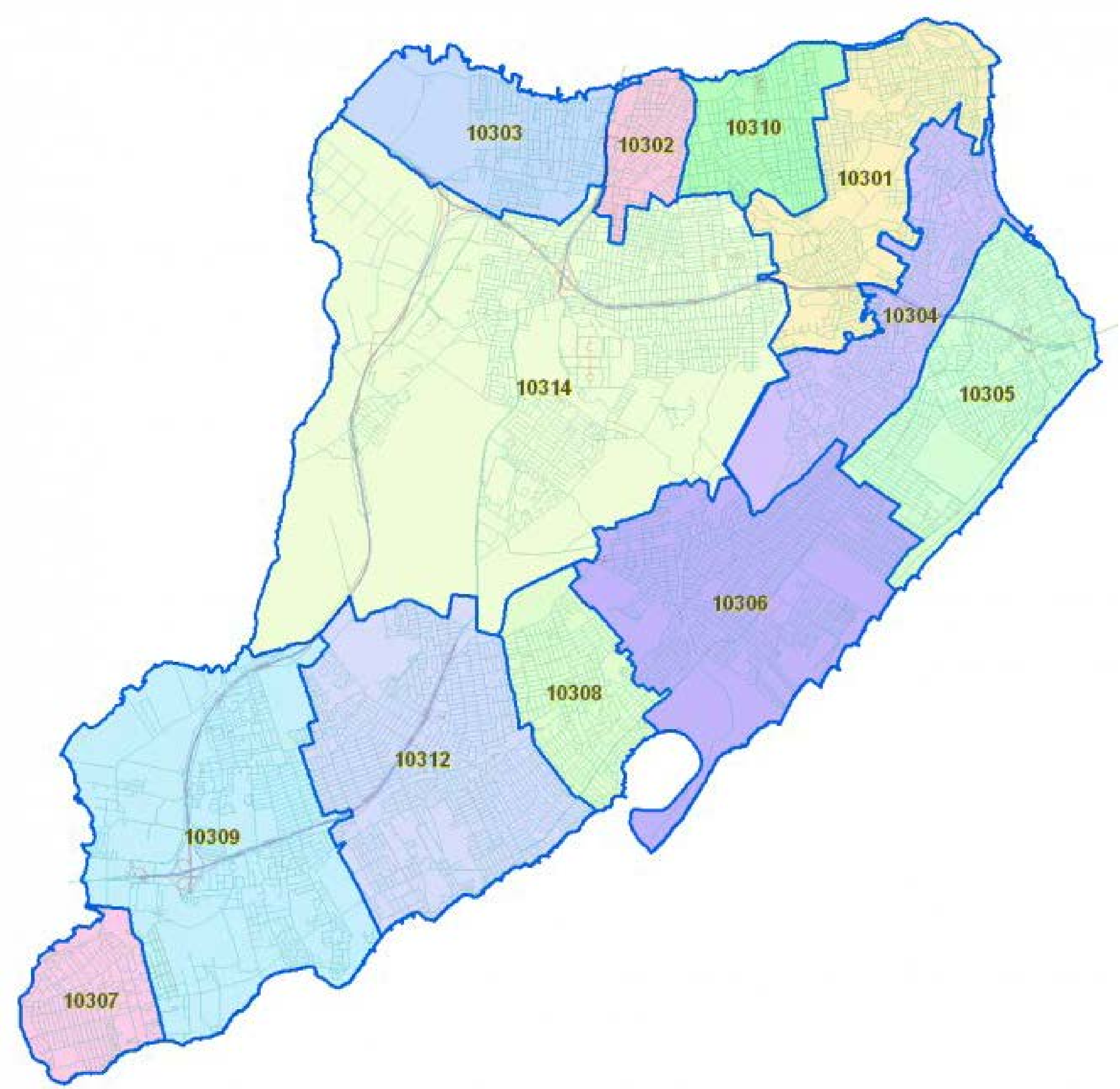

Supplement: S2 Appendix — (PDF) [file pone.0194799.s002.pdf]
